# Design and Application of a Flexible Blood Oxygen Sensing Array for Wearable Devices

**DOI:** 10.3390/mi13101742

**Published:** 2022-10-14

**Authors:** Wen-Cheng Kuo, Tzu-Chien Wu, Jun-Sheng Wang

**Affiliations:** Department of Mechatronics Engineering, National Kaohsiung University of Science and Technology, No. 2 Jhuoyue Rd., Nanzih, Kaohsiung 811532, Taiwan

**Keywords:** parylene, blood oxygen, sensing array, wearable device

## Abstract

The performance of portable or wearable oximeters is affected by improper movement or wear, which causes an error in the blood oxygen concentration calculation. The error comes from external incident stray light or light leakage caused by the improper fit of the sensor to the skin. This study aimed to develop a flexible blood oxygen sensing system with a 3 × 3 array that uses a reflective-type blood oxygen sensing chip to sequentially measure the blood oxygen levels at nine locations through a time division pulse modulation method. Each sensing chip has light transmission and receiving parts. A flip chip package was used to integrate the sensing chip, and a flexible parylene substrate that could fit the curvature of the wrist and locate the array of photo diodes around the radial artery of the wrist was used. By scanning the sensor array in dynamic behavior, the correct light intensity can be extracted to obtain the blood oxygen concentration and prevent errors due to improper fit or sensor movement during exercise.

## 1. Introduction

Blood oxygen sensing is currently widely used in medical applications for the immediate monitoring of human blood oxygen levels. Oxygen saturation directly reflects the oxygen supply capacity of blood, and oxygen is one of the primary factors for the aerobic metabolism of cell tissue. Hypoxia is harmful to human physiology and can be fatal.

An oxygen saturation percentage higher than 90% is considered normal. A score of 90% or less [1] indicates an aberration that must be further investigated to determine the presence of any undetected health concerns.

**Figure 1:**
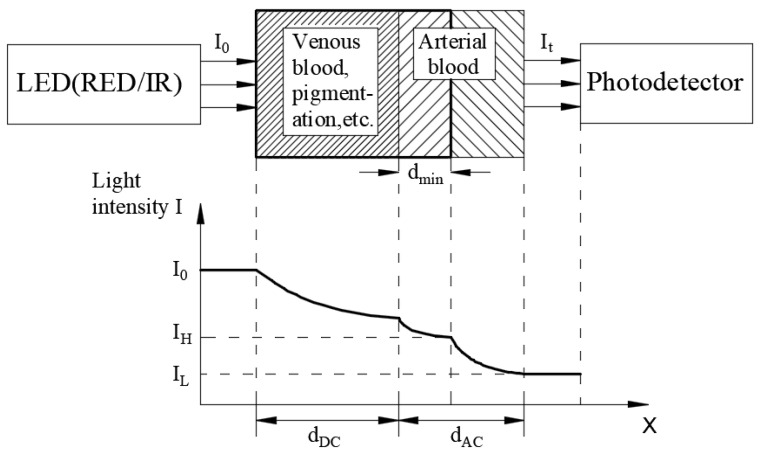
Illustration of the Beer–Lambert law.

The early medical equipment was very large and impractical. In recent years, there have been some analysis methods of physiological signals such as the use of ECG [2] and photoplethysmography (PPG) [3,4] waveforms. The principle of biological radar detection is commonly used to analyze the ECG waveform [2], and the Beer–Lambert law [5,6] is commonly used to analyze the PPG waveform. PPG has been widely implemented in blood oxygen sensors. The concept of PPG is to measure the characteristics of the light absorption intensity when the light penetrates the skin and tissue, calculating the blood oxygen value through the use of red and infrared light that penetrates the biological tissue. When light travels through biological tissues, it is absorbed by various tissue absorption substances such as skin colors, bones and muscles, venous blood, and arterial blood and the arterial blood vessel diameter changes with heart contraction and relaxation. As depicted in Figure 1, the Beer–Lambert law states that the components that cause light attenuation are divided into two categories: alternating signal (AC) and direct current signal (DC). The composition changes from *I_H_* (maximum value) to *I_L_* (minimum value). AC refers to the heart pulse and changes, whereas DC refers to the components that do not change with heart pulsation. The amount of light absorbed by DC is fixed. Nowadays, numerous people are using these wearable devices to determine their current physical condition and monitor their health. Blood oxygen measuring instruments have gradually developed from large-scale device systems to small wearable blood oxygen measuring devices.

In optical blood oxygen measurement methods, PPG has numerous advantages such as a small size, low power consumption, less dependency on a power supply, less electromagnetic interference, and a rapid response. Therefore, it has been gradually introduced into various applications including fitness trackers and homecare equipment.

PPG oximeters can be divided into two categories: transmitting and reflective oximeters, depending on the location of the light emitting and received signals. The light-emitting components (light-emitting diode (LED)) and -receiving components (photodiodes) of the transmitting oximeter are located on both sides of the measuring object. Furthermore, the light-emitting and -receiving parts of the reflective oximeter are located on the same side of the measuring object, there is a greater number of measuring positions, and the device is convenient to wear. The key advantage of the reflective blood oxygen sensor is convenience, which enables the device to be applied in the growing homecare market, and thus many of these wearable devices are available in the market. Depending on the measurement position, these can be divided as follows: ear-clip-type oxygen measurement [7,8], forehead oxygen measurement [7,9], and watch pulse oximetry [10].

Ear-clip-type blood oxygen sensors are easy to manufacture and are widely used in pulse oximetry. Spring-type ear clips were used in early designs. Although it is feasible to use them for measurement, its clamping pressure hinders operations during long-term measurement. A new type of microelectromechanical system (MEMS) technology can be used to create a lighter and more comfortable earphone oximetry device, which enables the monitoring of the ear oximetry sensor during physical activities [7].

The advantages of forehead oxygen measurement compared with other measurements in the body are that the thin cortex of the forehead and prominent skeletal structure help to redirect the light to the photodiode, thus providing a higher sensitivity and accuracy to changes in arterial signals [7] and reducing errors caused by physical activity. A forehead measurement device, which is generally installed on a helmet or sports headscarf, can be used during physical activity or to inform other soldiers of their current physiological condition on the battlefield [9].

In watch-type pulse oximetry, most wearable sports watch devices are used to measure the pulse and heart rate. However, because of the problem of light leakage, they are rarely used to measure the human blood oxygen concentration in dynamic behavior. A new PPG oximetry module (e.g., those of Garmin and Apple’s iWatch) with a watch-type design has been developed as a wearable device to measure the blood oxygenation of the ulnar artery of the radial artery and wrist. Although these devices are easy to wear, they are not conventionally used for clinical purposes [10].

Improper site selection or sensor movement causes inaccurate light intensity reception by the sensor, and this generates the blood oxygen saturation concentration (*S_p_O*_2_) error. Limited by the width and curvature of the wrist, it is not possible to install too many arrays. Too many arrays would increase the size of the devices, and the outer sensor chips would be far away from the radial artery of the wrist, causing measurement errors. This research developed a 3 × 3 reflective sensing array for flexible wrist pulse oximetry to obtain *S_p_O*_2_. Each sensing chip has individual transmission (LED) and receiving (photodiode) components. The flip chip package was used to integrate the sensing chips, and a flexible parylene substrate that could fit the curvature of the wrist and locate the array of LEDs and photodiodes around the radial artery of the wrist was used. By scanning the sensor array, the correct light intensity can be extracted to obtain *SpO*_2_ and prevent errors due to improper fitting or sensor movement during exercise.

## 2. Research Method and Design

**Figure 2:**
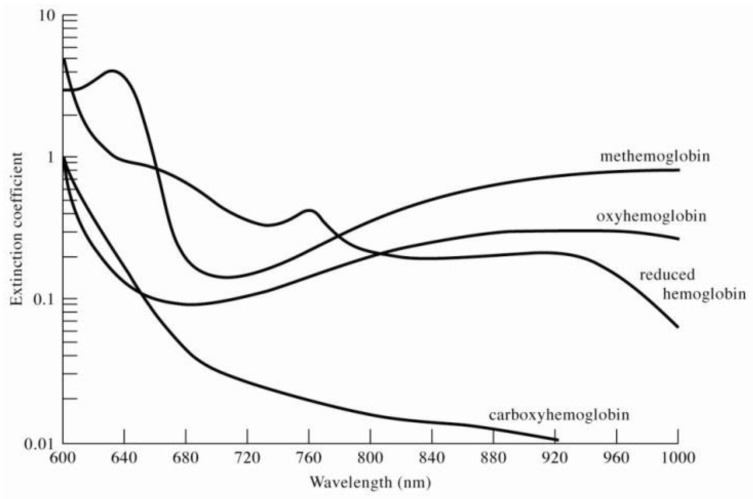
The light absorbance spectra for methemoglobin, oxyhemoglobin, reduced hemoglobin, and carboxyhemoglobin.

To evaluate *S_p_O*_2_ in human veins or arteries, the blood oxygen saturation concentration was determined by measuring the difference in the ratio of oxygen in the light absorption spectrum to the difference in the absorption spectrum of oxygenated hemoglobin (*H_b_O*_2_) and deoxygenated hemoglobin (*H_b_*). Figure 2 presents the light absorbance spectra for oxygenated hemoglobin and deoxygenated hemoglobin [11].

The Beer–Lambert law concerns the attenuation of light and the properties of the material through which the light travels [5]. As a solution, for a certain wavelength of light absorbed by the medium in the solution, the transmitted light penetrating the solution follows the medium in the solution. The intensity of light transmitted through a tissue that includes vessels with whole blood is represented as follows [12]:

*I_t_* = *I*_0_exp(−*ɛCl*), *In*(*I*_0_/*I_t_*) = *ɛCl* (1)

where *I_t_*, *I*_0_, *ɛ*, *C*, and *l* represent the transmitted light intensity, incident light intensity, an extinction coefficient, the concentration of hemoglobin in the tissue, and the path of light, respectively.

The incident light intensity *I*_0_ of the light that passes through the tissue to be measured is affected by components such as venous blood, arterial blood, skin, bones, and other tissues (DC component), which attenuate light intensity and are pulsed in the blood. *I_L_* is lower light transmission through the tissue at a higher tissue blood volume, and *I_H_* is higher transmitted light at a lower tissue blood volume. Therefore, Equation (1) can be rewritten as follows:

*I_L_* = *I_H_*exp(−*ɛ*Δ*Cl*), ln(*I_H_*/*I_L_*) = *ɛ*Δ*Cl* (2)

Minor changes to blood vessels are expressed as (*I_H_* − *I_L_* << *I_L_*) and *ln*(*I_H_*/*I_L_*) is approximate to (*I_H_* − *I_L_*)/*I_L_*, and thus Equation (2) can be expressed as follows:

(*I_H_* − *I_L_*)/*I_L_* = *ɛ*Δ*Cl* (3)

**Figure 3:**
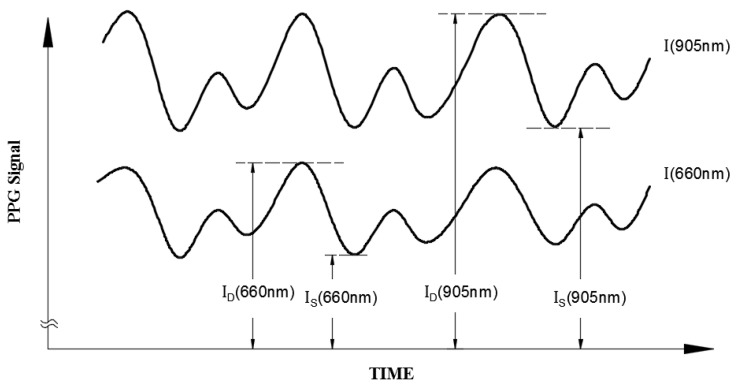
Illustration of the separated red (660 nm) and infrared (905 nm) patient signals with their I_D_ and I_S_ values caused by arterial pulsation.

For the blood oxygen concentration measurement (Figure 3), this study was conducted using a red light source (660 nm) and infrared (905 nm) dual-wavelength light source, and light from these sources was transmitted into the human tissue. In Equation (3), *I_d_* and *I_s_* are substituted for *I_H_* and *I_L_*, respectively. The light absorption ratio (R) at the two wavelengths can be defined as follows:

(4) $$R=\frac{{\left[\left(I_{H}-I_{L}\right)/I_{L}\right]}_{\left(660\right)}}{{\left[\left(I_{H}-I_{L}\right)/I_{L}\right]}_{\left(905\right)}}=\frac{{\left[\left(I_{D}-I_{S}\right)/I_{S}\right]}_{\left(660\right)}}{{\left[\left(I_{D}-I_{S}\right)-I_{S}\right]}_{\left(905\right)}}$$

The blood oxygen saturation concentration (*S_p_O*_2_) equation [13], where *H_b_O*_2_ and *H_b_* represent the concentrations of oxygenated hemoglobin in the blood and no hemoglobin in the blood, is defined as follows [14]:

(5) $$S_{P}O_{2}=\frac{H_{b}O_{2}}{H_{b}O_{2}+H_{b}}\times 100\%$$

The extinction coefficient *ɛ* of the hemoglobin is defined as the absorption constant of the blood sample divided by the concentration of the hemoglobin in the sample. The total extinction coefficient *ɛ* of the hemoglobin in the blood sample is related to its oxygen saturation because the hemoglobin in blood is a mixture of *HbO*_2_ and *Hb* with the extinction coefficients $\varepsilon_{0}$ and $\varepsilon_{D}$, respectively, and can be represented as follows:

(6) $$\varepsilon =\varepsilon_{0}S_{P}O_{2}+\varepsilon_{D}\left(1-S_{P}O_{2}\right)=\varepsilon_{D}+S_{P}O_{2}\left(\varepsilon_{0}-\varepsilon_{D}\right)$$

If the difference between the blood concentration change Δ*C* in the two wavelengths is neglected, this satisfies Equation (6), and R can be approximately expressed as follows [12]:

(7) $$R\approx \varepsilon_{\left(660\right)}/\varepsilon_{\left(905\right)}$$

The relationship between the measured parameter *R* and *SpO*_2_ in the arterial blood can be derived using Equations (6) and (7) through a simple manipulation as follows:

(8) $$S_{P}O_{2}=\frac{\epsilon_{D\left(660\right)}-R\cdot \varepsilon_{D\left(905\right)}}{R\left(\varepsilon_{0\left(905\right)}-\varepsilon_{D\left(905\right)}\right)+\left(\varepsilon_{D\left(660\right)}-\varepsilon_{D\left(660\right)}\right)}$$

Equation (9) was calculated using the values of *ɛ_D_* and *ɛ*_0_ in Table 1 [11,15], presenting the relationship between the oxygen concentration value and R value.

(9) $$S_{P}O_{2}=\frac{0.81-0.21\times R}{0.09\times R+0.73}$$

**Figure 4:**
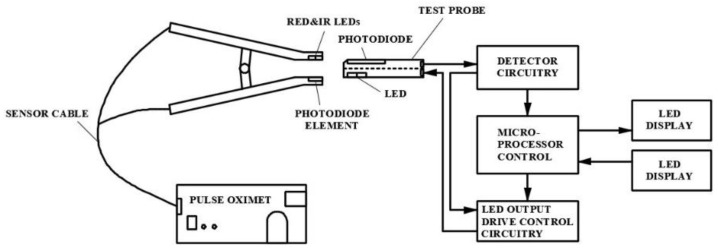
Schematic diagram of the LED emitter and photodiode calibrated by the pulse oximeter simulator.

**Figure 5:**
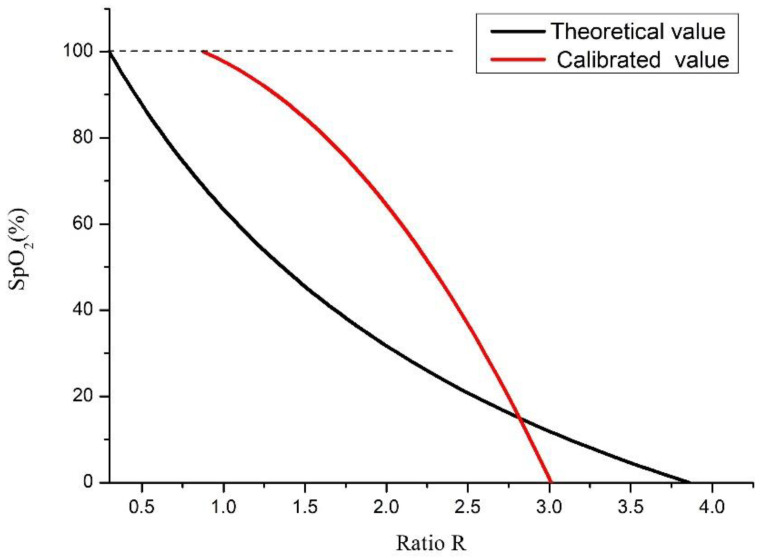
Relationship between the oxygen concentration value and R value.

However, Equation (9) is the theoretical *SpO*_2_ value calculated using the R value. In practical applications, the performance of the LED emitter and photodiode needs to be calibrated to obtain the correct value. In this study, the FLUKE Index 2 Pulse Oximeter Simulator was used as the calibration instrument [16]. The test probe of the FLUKE Index 2 Pulse Oximeter Simulator detects the red light and infrared light emitted by the LED emitters, and then uses the LED emitter of the test probe to emit the red and infrared light by simulating 35–100% of the *SpO*_2_ value to correct the photodiode used in this study and control the error rate within 1%. The calibration method is shown in Figure 4. The LED emitters used in this study emit red light (Red) and infrared light (IR), which are received by the photodiode on the test probe. The programmed calibrated default *SpO*_2_ of the PPG waveform is sent to the LED emitter on the test probe to emit, and is received by the photodiode used in this study. By calculating the peak and trough values of the red light and infrared light waveforms, the corrected R value under the corresponding *SpO*_2_ value can be obtained. Figure 5 shows the relationship between the theoretical and corrected blood oxygen saturation concentration values in relation to the R value.

In addition to the fact that the LED and photodiode must be calibrated, the emitter and receiver chips must be properly attached to the skin to avoid receiving external incident light and signaled light leakage, resulting in errors in *SpO*_2_ measurement. This study developed a sensing system with a 3 × 3 array to sequentially measure the blood oxygen levels at nine locations through a time division pulse modulation method (TDPM).

If the LED emitter and receiver are not properly fitted with the skin, the external incident stray light will be received by the receiver, and some of the light emitted by the LED emitter cannot be received since it is too large (external incident stray light) or too small (signal light), and these wrong PPG waveforms are easy to identify. In real cases, it is not easy to generate a complete PPG waveform. In addition, compared to the normal received light intensity, the light intensity is higher than expected. These factors make it easy to identify incorrect R values and blood oxygen values. When the tester exercises a dynamic behavior, the sensing system can receive the PPG waveforms received by all receivers for calculation and extract the correct blood oxygen value; if one receiver can receive the signal correctly, it can provide the correct *SpO*_2_ value.

## 3. Fabrication

A 3 × 3 array blood oxygen sensing system based on the PPG method and Beer–Lambert law was proposed that uses a reflective blood oxygen sensing module to sequentially measure the blood oxygen levels at nine locations.

**Figure 6:**
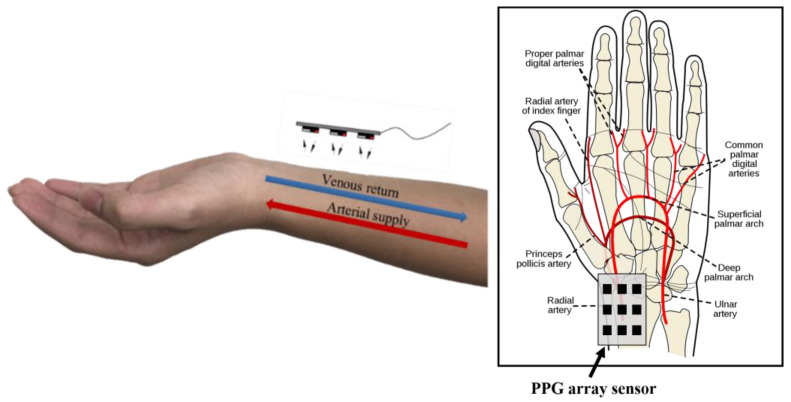
Illustration of the radial artery and sensing array location.

The measurement position was on the radial artery of the left hand [17] (Figure 6). To increase the closeness between the sensor and skin on the curvature of the wrist, a biocompatible material (parylene) was used as the flexible substrate on which to fabricate the flexible circuit board that was packaged with nine commercial oxygen concentration sensing chips (DCM-05, AMPKorea; specifications shown in Table 2) using the flip chip technique. The sensing module chip can be attached to the skin for blood oxygen concentration measurement.

**Figure 7:**
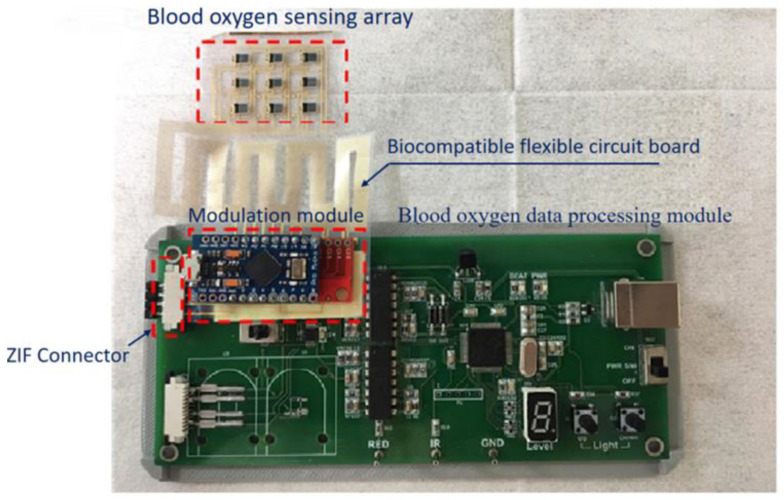
The actual blood oxygen sensing system.

The proposed flexible blood oxygen sensing array system developed in this research can be divided into five categories: a blood oxygen sensing array, a modulation module, a blood oxygen data processing module, a zero-insertion-force (ZIF) [18] connector, and a biocompatible flexible circuit board (Figure 7).

### 3.1. Blood Oxygen Sensing Array

**Figure 8:**
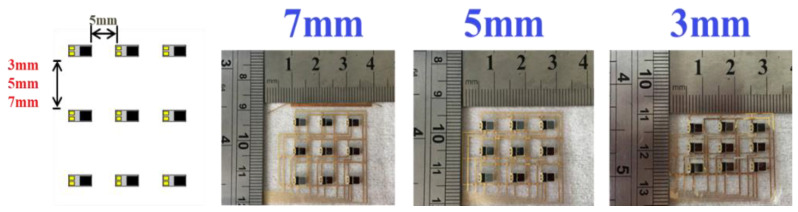
Three sensing element spacing designs: 3 mm, 5 mm, and 7 mm.

In this study, sensing chips were placed in a 3 × 3 array. The length and width of the array area were 25 and 18 mm, respectively. The distances between the sensing chips were 3 mm, 5 mm, and 7 mm (Figure 8). When the device was smoothly attached to the skin, the accurate PPG waveform could be measured, and *SpO*_2_ could be obtained.

### 3.2. Modulation Module

**Figure 9:**
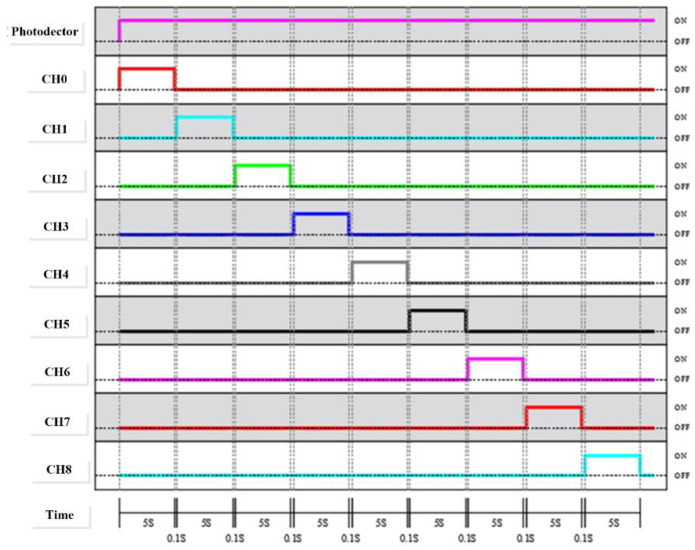
Time table of the photo detector and sensing element actuation.

The processing method for the data from the 3 × 3 sensing module (Ch0~Ch8) was based on the time division pulse modulation (TDPM) method (Figure 9), where the red and infrared light emitted by each channel were sequentially received by the photodetector. In this study, the TDPM was used to perform the sweep measurement of nine sensing chips, the sensing time of each channel was 5 s, and the switching time was 0.1 s. The modulation module comprised an Arduino Micro Development Board (Arduino Micro; specifications shown in Table 3) and a multiplexer CD74HC4067 (specifications shown in Table 4) that were used to perform and execute the TDPM. The Arduino Micro sent digital commands to control the CD74HC4067, causing it to perform a sweep, guiding the analog signals from nine channels to the blood oxygen data processing module.

### 3.3. Blood Oxygen Data Processing Module

**Figure 10:**
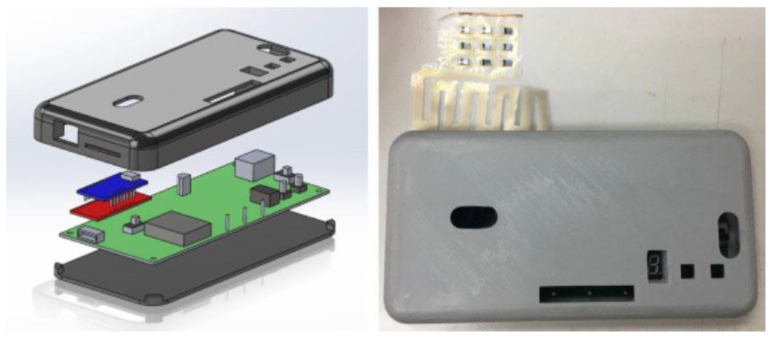
Illustration of the real 3D printing case.

The blood oxygen data processing module (SDPPG Kit, AMPKorea Inc.) received the analog PPG signals from nine channels to record and change the analog signals into digital signal data points. To integrate the modulation module and blood oxygen data processing module to yield a portable device, the Solidworks software was used to design the device case (Figure 10), and 3D printing technology was used to create it.

### 3.4. ZIF Connector

Wire bonding is a conventional method for connecting the sensing chip and circuit board; however, the parylene substrate in this study could not withstand high-temperature soldering. To ensure the smooth connection between the sensor chip and parylene flexible circuit board, the ZIF connector [18] was used to connect the parylene flexible substrate and printed circuit board.

### 3.5. Biocompatible Flexible Circuit Board

**Figure 11:**
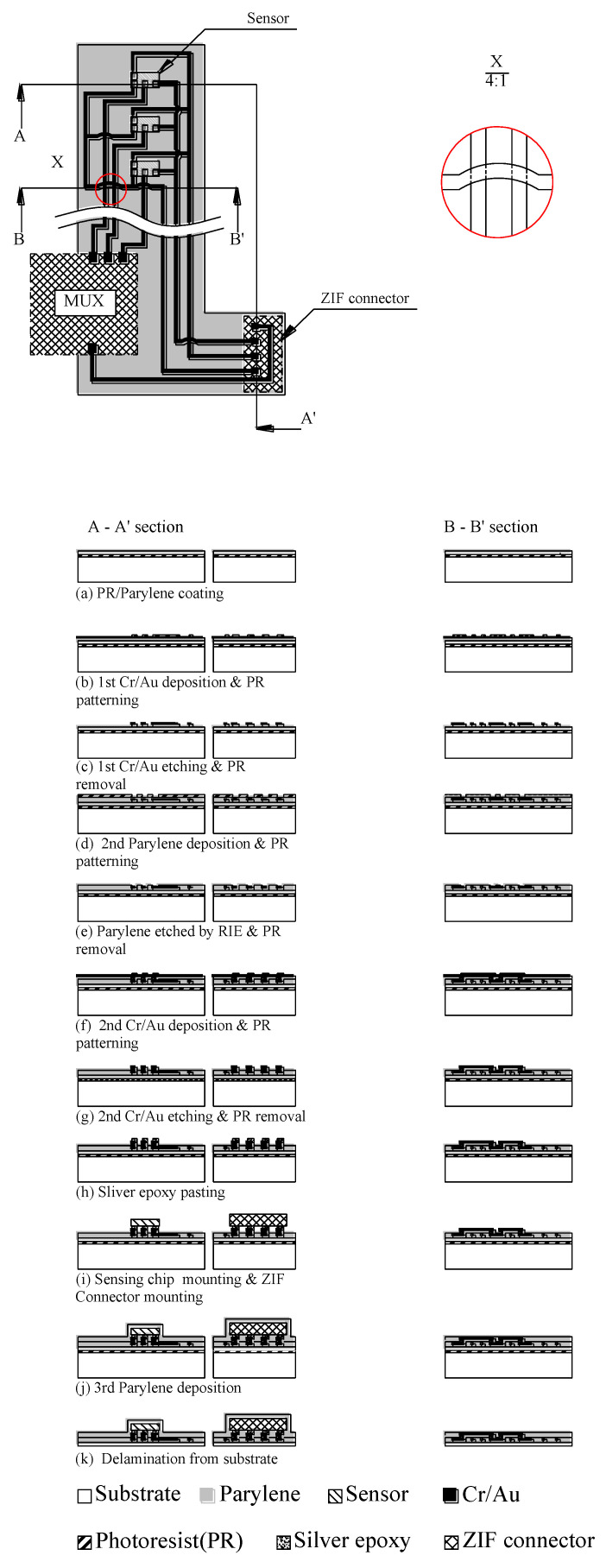
The fabrication process of the biocompatible flexible blood circuit board.

This study used a biocompatible grade of parylene as the flexible substrate, and gold was then deposited as conduction wires and pads to connect the sensing chip. The flip chip packaging technology was used to fit the sensing chips on the pads. The biocompatible flexible circuit board enabled the attachment of sensing chips and the skin to measure the blood oxygen concentration. Figure 11 shows the fabrication process of the biocompatible flexible circuit board, in which there are two cross-sections, A-A’ and B-B’. A-A’ represents the sensor and ZIF contacts, respectively, and B-B’ represents the interconnection/jumper wire. The process steps are described stepwise as follows:

Step (a): PR/parylene coating

The photoresist (PR) was coated on the substrate using a spin coater, and the biocompatible parylene film was then deposited on the glass substrate through chemical vapor deposition. As a flexible substrate, its thickness was 30 μm.

Step (b): First Cr/Au deposition and PR patterning

Cr and Au were deposited through sputtering with a thickness of 20/200 nm to create a seed layer and the conduction wires, respectively. The unetched area was defined by photoresist AZ-4620 with a thickness of 5 μm.

Step (c): First Cr/Au etching and PR removal

Unprotected Au and Cr were etched using Au etchant and Cr-7, respectively. PR was removed using acetone.

Step (d): Second parylene deposition and PR patterning

A parylene film was deposited through chemical vapor deposition to protect and insulate the inner gold wire with a thickness of 5 μm. The etched pattern was defined by the photoresist AZ-4620 for the purpose of via opening.

Step (e): Parylene etched using reactive ion etching and PR removal

The unprotected parylene by the photoresist above the via opening was etched using reactive ion etching (RIE) and oxygen plasma to expose the via, and the photoresist was removed using acetone.

Step (f): Second Cr/Au deposition and PR patterning

Using sputtering, Cr and Au were deposited with a thickness of 20/200 nm to create the seed layer and second conduction wires, respectively. The unetched area was defined through photoresist AZ-4620 with a thickness of 5 μm.

Step (g): Second Cr/Au etching and PR removal

Unprotected Au and Cr were etched using the Au etchant and Cr-7, respectively. The photoresist was removed using acetone.

Step (h): Silver epoxy pasting

A biocompatible grade of silver was pasted on the gold connection pads for the flip chip mounting of sensing chips.

Step (i): Sensing chip mounting and ZIF connector mounting

After the silver glue was coated on the gold connection pads, nine sensing chips and ZIF connectors were attached to the gold connection pads, and were then baked in an oven at 80 °C for 3 h.

Step (j): Third parylene deposition

The third parylene deposition was applied to create the upper protection layer to protect and insulate the inner parts.

Step (k): Delamination form substrate

The biocompatible flexible circuit board was released from the substrate by peeling.

**Figure 12:**
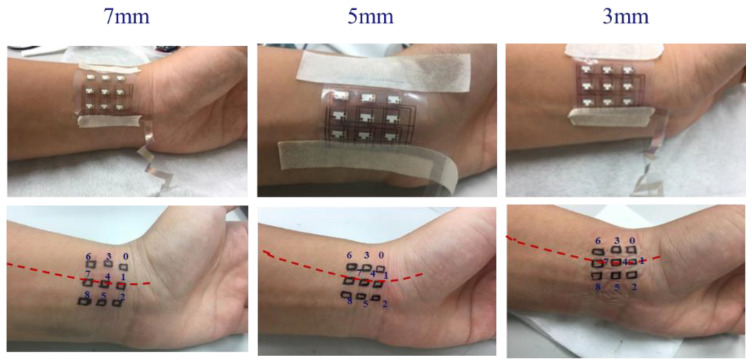
Photos of the sensing chip number and placement of three different pitch sensing chips. The red dotted line indicates the radial artery position and crosses the center of the sensing chips.

Three designs were proposed (Figure 8) for the sensing chip array location. The spacing in the x-direction was fixed at 5 mm, and the spacings in the y-direction were 3 mm, 5 mm, and 7 mm, respectively. Figure 12 depicts photos of the sensing chip number and location of three different pitch sensing arrangements. The red-dotted line indicates the radial artery position and crosses the center of the sensing chips (Nos. 1, 4, and 7).

## 4. Measurements

In this study, the blood oxygen sensing array was used for dynamic measurements to examine the influence of the light leakage phenomenon caused by the improper fit between the sensor and the skin in the dynamic behavior of blood oxygen detection. To take a dynamic measurement, the arm was slightly swung to simulate the state of people walking during an activity. The arm swing frequency was approximately 70 times per minute. The arm was swung once to represent one step. A person walked one step and moved a distance of approximately 70–80 cm. A simulated speed of 3.15 km/h or 19.04 min/km was used to evaluate the feasibility of the device in the study. The measurement results indicate the sensor movement caused by the swing of the arm.

**Figure 13:**
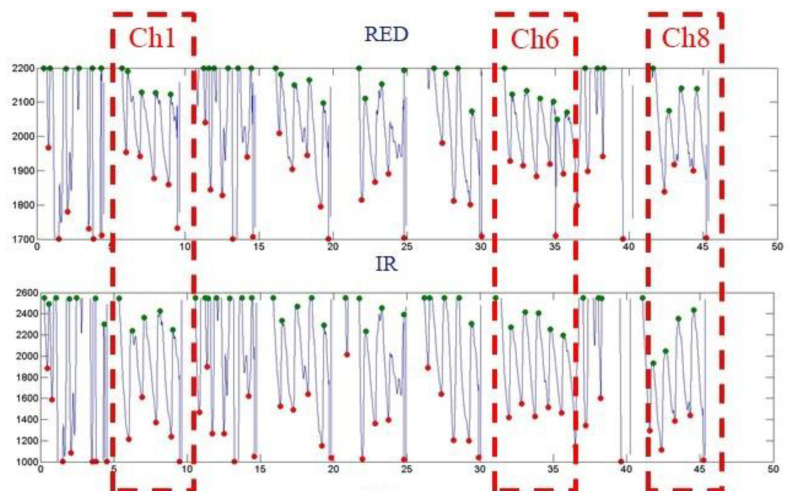
Dynamic state of the blood oxygen concentration value measurement results of a flexible blood oxygen sensing array with a pitch of 3 mm.

**Figure 14:**
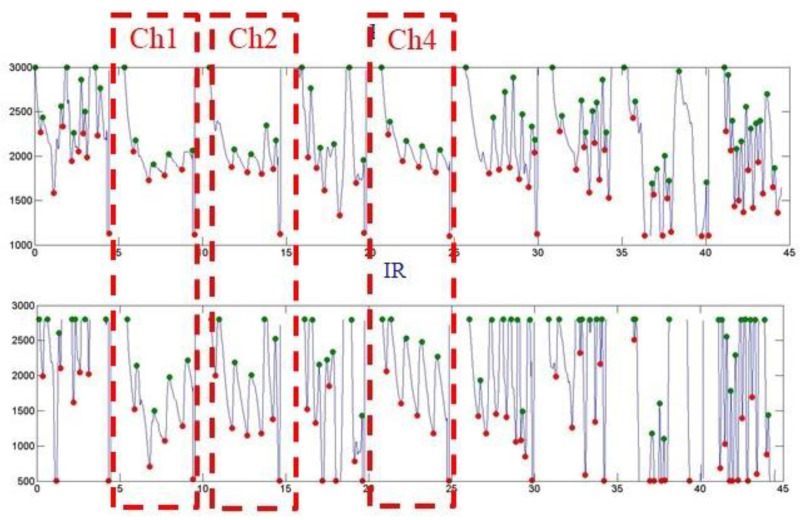
Dynamic state of the blood oxygen concentration value measurement results of a flexible blood oxygen sensing array with a pitch of 5 mm.

**Figure 15:**
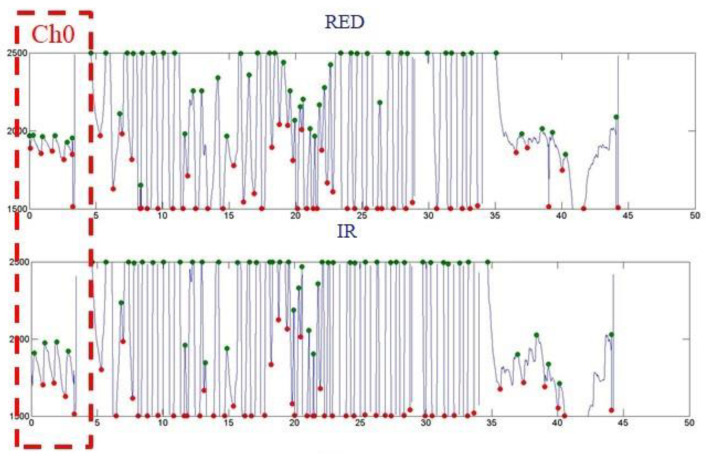
Dynamic state of the blood oxygen concentration value measurement results of a flexible blood oxygen sensing array with a pitch of 7 mm.

Figure 13, Figure 14, and Figure 15 show the PPG waveforms of the blood oxygen array design with spacings of 3, 5, and 7mm, respectively. It can be found in these PPG waveforms that due to the influence of improper fitting, the intensity of the external incident stray light is greater than the intensity of the light received by the emitter, and it is difficult to form a complete PPG waveform. It can be determined that it is not a correct PPG waveform. Figure 13 shows that only Ch1, 6, and 8 presented complete PPG waveforms under the design with a sensor spacing of 3 mm. Figure 14 shows that only Ch1, 2, and 4 presented complete PPG waveforms under the design with a sensor spacing of 5 mm. Figure 15 shows that only Ch0 presented complete PPG waveforms under the design with a sensor spacing of 5mm. Comparing the complete PPG waveforms in Figure 13, Figure 14, and Figure 15, the received light intensity of the 7 mm spacing design was smaller than that of the 3 and 5 mm designs because the sensor was far from the radial artery, so the received signal of the radial artery was relatively small.

In the calculation of the blood oxygen concentration, if there are multiple sensors that receive the correct PPG waveform, and because the abnormal reception will reduce the light intensity, the R value with the largest light intensity was selected for the calculation of the blood oxygen concentration. Table 5, Table 6 and Table 7 show the calculated blood oxygen values of the array sensors under the design with sensor spacings of 3, 5, and 7 mm, respectively. From these tables, it can be seen that the values of the blood oxygen values calculated from the complete PPG waveform were very close. The reason for this is that the R value is the ratio of the intensity of red light to infrared light. If the received intensities of red light and infrared light are both attenuated to the same degree, the difference in the ratio is not significant.

## 5. Conclusions

This study proposed a 3 × 3 array blood oxygen sensing system based on the PPG method and Beer–Lambert law that uses a reflective blood oxygen sensing module to sequentially measure the blood oxygen levels at nine locations. A flexible parylene based substrate to keep the sensor fit to the skin can reduce the blood oxygen concentration calculation error caused by light leakage. The dynamic behavior measurement results show that the smaller the spacing of sensors away from the radial artery, the more sensors that can receive the complete PPG waveform, the greater the received light intensity. The array type sensor design can detect the blood oxygen concentration value at multiple points. Under dynamic behavior, one sensor is correctly attached to the skin, and the blood oxygen concentration value can be obtained.

## Figures and Tables

**Table 1:** The extinction coefficients (Lmmol^−1^cm^−1^) of the reduced (*εH_b_*) and (*εH_b_O*_2_) oxygenated hemoglobin at the wavelengths of 660 nm and 905 nm.

Wavelength (nm)	*εH_b_*	*εH_b_O*_2_
660	0.81	0.08
905	0.21	0.30

**Table 2:** The datasheet of DCM05.

SYMBOL	CHARACTERISCTIC	COMPONENTS	TEST CONDITION	MIN	TYP	MAX	UNITS
VF	Forward Voltage	LED1	IF = 20 mA	-	1.9	2.2	V
		LED2		-	1.3	1.5	
		PD	IF = 10 mA	0.5	-	1.3	
IR	Revers Breakdown Current	LED1, LED2	VR = 5 V	-	-	10	uA
PO	Output Power	LED1	IF = 20 mA	-	2	-	mW
		LED2		-	2	-	
λ PEAK	Peak Wavelength	LED1	IF = 20 mA	-	660	-	nm
		LED2		-	905	-	
		PD		-	940	-	
Δλ	Half Wave Width	LED1	IF = 20 mA	-	30	-	nm
Δλ V BR	Half Wave Width Reverse Breakdown Voltage	LED2	IF = 20 mA IR = 100 Ua	-	60	-	nm V
		PD		35	-	-	
I D	Reverse Dark Current	PD	V R = 10 V	-	-	20	nA
I L	Light Current		1mW@940 nm	-	21	-	uA
S	Spectral Response Range		-	400	-	1050	nm
CJ	Junction Capacitance		VR = 3 V, f = 1 MHz	-	20	-	pF

**Table 3:** Datasheet of the Arduino Micro Development Board.

Dimension	35 mm × 18.5 mm × 4.5 mm
Microcontroller	ATmega32u4
Operating Voltage	5 V
Input Voltage (recommended)	7–12 V
Digital I/O Pins	20
PWM Channels	7
Analog Input Channels	12
DC Current for 5V Pin	40 mA
DC Current for 3.3V Pin	50 mA
Flash Memory	32 KB (ATmega32u4) of which 4 KB used by bootloader
SRAM	2.5 KB
EEPROM	1 KB
Clock Speed	16 MHz

**Table 4:** Datasheet of the high-speed CMOS 16-channel analog multiplexer/demultiplexer CD74HC4067.

PARAMETER	SYMBOL	TEST CONDITIONS		VCC (V)	25 °C			−40 °C TO 85 °C		−55 °C TO 125 °C		UNITS
		VI (V)	VIS (V)		MIN	TYP	MAX	MIN	MAX	MIN	MAX	
HC TYPES												
High Level Input Voltage	V_IH_	-	-	2	1.5	-	-	1.5	-	1.5	-	V
				4.5	3.15	-	-	3.15	-	3.15	-	V
				6	4.2	-	-	4.2	-	4.2	-	V
Low Level Input Voltage	V_IL_	-	-	2	-	-	0.5	-	0.5	-	0.5	V
				4.5	-	-	1.35	-	1.35	-	1.35	V
				6	-	-	1.8	-	1.8	-	1.8	V
Maximum “ON” Resistance I_O_ = 1 mA	R_ON_	VCC or GND	VCC or GND	4.5	-	70	160	-	200	-	240	Ω
				6	-	60	140	-	175	-	210	Ω
		VCC to GND	VCC to GND	4.5	-	90	180	-	225	-	170	Ω
				6	-	80	160	-	200	-	240	Ω
Maximum “ON” Resistance Between Any Two Switches	ΔR_ON_	-	-	4.5	-	10	-	-	-	-	-	Ω
				6	-	8.5	-	-	-	-	-	Ω
Switch “Off” Leakage Current 16 Channels	I_IZ_	Ē = VCC	VCC or GND	6	-	-	±0.8	-	±8	-	±8	μA
Logic Input Leakage Current	I_I_	VCC or GND	-	6	-	-	±0.1	-	±1	-	±1	μA
Quiescent Device Current I_O_ = 0 mA	I_CC_	VCC or GND	-	6	-	-	8	-	80	-	160	μA

**Table 5:** The calculated blood oxygen concentration of the array sensors using the design with a sensor spacing of 3 mm.

Ch0	Ch3	Ch6
Ave. *S_p_O*_2_ N.A	Ave. *S_p_O*_2_ N.A	**Ave. *S_p_O*_2_** **~99**
**Ch1**	Ch4	Ch7
**Ave. *S_p_O*_2_** **~76**	Ave. *S_p_O*_2_ N.A	Ave. *S_p_O*_2_ N.A
Ch2	Ch5	**Ch8**
Ave. *S_p_O*_2_ N.A	Ave. *S_p_O*_2_ N.A	**Ave. *S_p_O*_2_** **~99**

**Table 6:** The calculated blood oxygen concentration of the array sensors using the design with a sensor spacing of 5 mm.

Ch0	Ch3	Ch6
Ave. *S_p_O*_2_ N.A	Ave. *S_p_O*_2_ N.A	Ave. *S_p_O*_2_ N.A
**Ch1**	**Ch4**	Ch7
**Ave. *S_p_O*_2_** **~99**	**Ave. *S_p_O*_2_** **~98**	Ave. *S_p_O*_2_ N.A
**Ch2**	Ch5	Ch8
**Ave. *S_p_O*_2_** **~99**	Ave. *S_p_O*_2_ N.A	Ave. *S_p_O*_2_ N.A

**Table 7:** The calculated blood oxygen concentration of the array sensors using the design with a sensor spacing of 7 mm.

Ch0	Ch3	Ch6
**Ave. *S_p_O*_2_** **~99**	Ave. *S_p_O*_2_ N.A	Ave. *S_p_O*_2_ N.A
Ch1	Ch4	Ch7
Ave. *S_p_O*_2_ N.A	Ave. *S_p_O*_2_ N.A	Ave. *S_p_O*_2_ N.A
Ch2	Ch5	Ch8
Ave. *S_p_O*_2_ N.A	Ave. *S_p_O*_2_ N.A	Ave. *S_p_O*_2_ N.A

## Data Availability

The data presented in this study are available upon request from the corresponding author. The data are not publicly available due to funders, and so cannot be made freely available.
